# Baseline clinical predictors of antitumor response to the PARP inhibitor olaparib in germline BRCA1/2 mutated patients with advanced ovarian cancer

**DOI:** 10.18632/oncotarget.17005

**Published:** 2017-04-10

**Authors:** Saeed Rafii, Charlie Gourley, Rajiv Kumar, Elena Geuna, Joo Ern Ang, Tzyvia Rye, Lee-May Chen, Ronnie Shapira-Frommer, Michael Friedlander, Ursula Matulonis, Jacques De Greve, Amit M. Oza, Susana Banerjee, L. Rhoda Molife, Martin E. Gore, Stan B. Kaye, Timothy A. Yap

**Affiliations:** ^1^ Drug Development Unit, The Institute of Cancer Research and The Royal Marsden NHS Foundation Trust, London, UK; ^2^ University of Edinburgh Cancer Research UK Centre, Edinburgh, UK; ^3^ University of California San Francisco, San Francisco, CA, USA; ^4^ Sheba Medical Centre, Ramat Gan, Israel; ^5^ Prince of Wales Cancer Centre, Randwick, Australia; ^6^ Dana-Farber Cancer Institute, Boston, MA, USA; ^7^ Oncologisch Centrum UZ Brussel, Brussels, Belgium; ^8^ Princess Margaret Cancer Centre, University Health Network, Toronto, Canada; ^9^ Gynae-Oncology Unit, Royal Marsden Hospital, London, UK

**Keywords:** PARP inhibitor, olaparib, BRCA, ovarian cancer, predictive biomarkers

## Abstract

**Background:**

The PARP inhibitor olaparib was recently granted Food and Drug Administration (FDA) accelerated approval in patients with advanced *BRCA1/2* mutation ovarian cancer. However, antitumor responses are observed in only approximately 40% of patients and the impact of baseline clinical factors on response to treatment remains unclear. Although platinum sensitivity has been suggested as a marker of response to PARP inhibitors, patients with platinum-resistant disease still respond to olaparib.

**Results:**

108 patients with advanced *BRCA1/2* mutation ovarian cancers were included. The interval between the end of the most recent platinum chemotherapy and PARPi (PTPI) was used to predict response to olaparib independent of conventional definition of platinum sensitivity. RECIST complete response (CR) and partial response (PR) rates were 35% in patients with platinum-sensitive versus 13% in platinum-resistant (p<0.005). Independent of platinum sensitivity status, the RECIST CR/PR rates were 42% in patients with PTPI greater than 52 weeks and 18% in patients with PTPI less than 52 weeks (p=0.016). No association was found between baseline clinical factors such as FIGO staging, debulking surgery, *BRCA1* versus *BRCA2* mutations, prior history of breast cancer and prior chemotherapy for breast cancer, and the response to olaparib.

**Methods:**

We conducted an international multicenter retrospective study to investigate the association between baseline clinical characteristics of patients with advanced *BRCA1/2* mutation ovarian cancers from eight different cancer centers and their antitumor response to olaparib.

**Conclusion:**

PTPI may be used to refine the prediction of response to PARP inhibition based on the conventional categorization of platinum sensitivity.

## INTRODUCTION

The identification of *BRCA1* and *BRCA2* (*BRCA1/2*) genes has greatly enhanced our knowledge of the DNA repair pathways involved in cancer progression and has led to the exploitation of the concept of synthetic lethality in cancer therapy [1, 2]. Poly(ADP-ribose) polymerase (PARP) inhibitors were the first to translate this theory into clinical practice and promise a new strategy in personalizing cancer therapy. It is now more than ten years since the publication of the first preclinical evidence of PARP inhibitor synthetic lethality in *BRCA1/2* mutation cancers. During this time, multiple clinical trials have investigated the clinical activity of different PARP inhibitors in a range of cancers. Olaparib (Lynparza; AstraZeneca) is the most extensively studied PARP inhibitor in patients with advanced *BRCA1/2* mutated ovarian cancer and has now received Food and Drug administration (FDA) and European Medicines Agency (EMA) regulatory approval in the relapsed and maintenance treatment settings, respectively.

The clinical activity of olaparib in patients with advanced germline *BRCA1/2* mutation ovarian cancer has been assessed in a number of phase I/II trials. Overall, Response Evaluation Criteria In Solid Tumors (RECIST) complete or partial responses (CR/PR) or Gynecologic Cancer InterGroup (GCIG) CA125 antitumor responses ranged between 33%-59%, with a clinical benefit rate (RECIST or GCIG CA125 responses and RECIST stable disease) of 46-52% [3–7]. The antitumor efficacy of olaparib in recurrent ovarian cancer is currently being assessed in the randomized phase III SOLO-3 trial, which compares olaparib monotherapy to physician's choice single-agent chemotherapy in patients with advanced *BRCA1/2* mutated ovarian cancer (NCT02282020).

While phase I/II clinical trials with olaparib have reported impressive objective response rates in patients with advanced *BRCA1/2* mutation ovarian cancer, not all patients achieve the same level of benefit to olaparib. This highlights the need to identify individual patient predictive biomarkers of response and resistance to guide treatment decisions. A number of translational clinical studies have now been initiated to identify molecular biomarkers of response to olaparib and other PARP inhibitors. Several studies are investigating the different molecular markers that might predict for antitumor response to olaparib and other PARP inhibitors beyond germline *BRCA1/2* mutations, including somatic *BRCA1/2* mutations, functional classifiers of homologous recombination deficiency, gene expression profiling and genomic scarring signatures [8–12].

Earlier observations from limited clinical data suggested a potential relationship between prior sensitivity to platinum-based chemotherapy and antitumor responses to olaparib [4]. This has led us to investigate the baseline clinical characteristics of patients with advanced *BRCA1/2* mutation ovarian cancer, including the effects of platinum sensitivity as potential factors that may be used in the prediction of patients benefit to olaparib. In addition, we assessed the association between antitumor response to olaparib treatment and progression-free survival (PFS) and overall survival (OS).

## RESULTS

### Baseline characteristics

A total of 108 patients with germline *BRCA1/2* mutation advanced ovarian cancer from eight cancer centers who had been treated with olaparib at a dose of 200mg BID or greater were included in our study (Table 1). The median age of patients at enrolment to clinical trials was 55 years (range 38-79 years). The majority of patients (83 of 108; 77%) had high grade serous ovarian carcinoma. Other ovarian cancer subtypes included endometrioid (8 of 108; 7.4%), clear cell (9 of 108; 8.2%) and unknown histological subtypes (8 of 108; 7.4%). Staging at diagnosis was available for 98 of 108 patients: FIGO stage I (1 of 108, 0.9%), II (10 of 108, 9.3%), III (76 of 108, 70.3%) and IV (11 of 108, 10.2%). FIGO staging was not available for 10 of 108 (9.3%) patients. Primary or interval surgical debulking data were available for 78 of 108 (72.2%) patients. 58 (53.7%) patients had optimal debulking, while 20 (18.5%) patients had suboptimal debulking surgery at diagnosis. No surgical data were available for 30 (27.8%) patients. The median number of prior lines of chemotherapy for ovarian cancer before commencing treatment with olaparib was 3 (range 1-10). Of 108 patients, 65 (60.1%) were conventionally defined as platinum-sensitive, while 38 (35.2%) were platinum-resistant at the time of starting olaparib. Platinum response status for 5 patients (4.6%) was not available. 77 of 108 (71%) patients had germline *BRCA1* mutations, while 31 of 108 (29%) patients had germline *BRCA2* mutations. Forty (38%) patients had a past history of breast cancer, of whom 20 (22.2%) received one previous line of chemotherapy for breast cancer.

**Table 1:** Baseline characteristics of patients with *BRCA1/2* mutation advanced recurrent ovarian cancer treated with olaparib

Baseline Criteria		N (%)
**Study subjects**		108
**Age, Year Median (Range)**		55 (38-79)
**Tumor type**	High grade serous	83 (77%)
	Endometrioid	8 (7.4%)
	Others	9 (8.2%)
	Unknown	8 (7.4%)
**Stage at diagnosis**	1	1 (0.9%)
	2	10 (9.3%)
	3	76 (70.3%)
	4	11 (10.2%)
	Unknown	10 (9.3%)
**Optimally debulked?**	Yes	58 (53.7%)
	No	20 (18.5%)
	Unknown	30 (27.8%)
***BRCA1/2* mutation**	*BRCA1*	78 (79%)
	*BRCA2*	30 (21%)
**Past Medical History of Breast Cancer**	Yes	40 (38%)
	No	68 (62%)
**Previous breast cancer chemotherapy**	Yes	20 (18.5%)
	No	88 (81.5%)
**Platinum status at enrolment to Olaparib**	Platinum-Sensitive	65 (60.1%)
	Platinum-Resistant	38 (35.2%)
	Unknown	5 (4.7%)

### Antitumor responses

Patients who only had one prior line of chemotherapy prior to receiving olaparib had significantly higher rates of RECIST CR/PR compared to those who received more than one prior line of treatment (p=0.005). No differences in RECIST CR/PR rates were noted between patients with germline *BRCA1* (27.6%) mutations versus those with *BRCA2* (27%) mutations (p=0.31). There was no association between having a past history of breast cancer or receiving prior chemotherapy for breast cancer, and achieving RECIST antitumor responses to olaparib (Table 2).

**Table 2:** Association between baseline characteristics and antitumor responses to olaparib

Characteristic			p
**Platinum Status**	**Plt-S (N=65)**	**Plt-R (N=38)**	
Median PTPI	68.7w	25.9w	p<0.0001
CR/PR to olaparib	23 (35%)	5 (13%)	p=0.02
**Prior lines of chemo**	**1 (N=15)**	**>1 (N=91)**	
CR/PR to olaparib	8 (53.3%)	20 (23.5%)	p<0.005
**Platinum status and PTPI**	**Plt-S >24w PTPI (N=58)**	**Plt-R <24w PTPI (N=20)**	
CR/PR to olaparib	20 (34.5%)	2 (10%)	p=0.04
**PTPI (independent of Plt status)**	**Pts with PTPI >52w (N=35)**	**Pts with PTPI <52w (N=59)**	
CR/PR to olaparib	15 (42%)	11 (18%)	p=0.016
**BRCA1/2 status**	**BRCA1 (N=77)**	**BRCA2 (N=31)**	
CR/PR to olaparib	21 (27.6%)	7 (27%)	p=0.31
**Breast cancer history**	**Prior BC (N=40)**	**Without (N=68)**	
CR/PR to olaparib	17 (26.5%)	11 (29%)	p= 0.76
**Breast cancer chemotherapy**	**Chemo (N=20)**	**None (N=88)**	
**CR/PR to olaparib**	4 (22.2%)	24 (28.5%)	p=0.64

**Figure 1:**
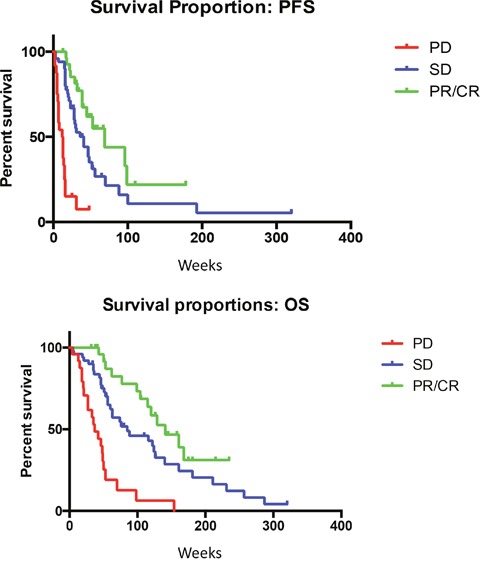
**(A)** Median PFS is significantly improved for patients with RECIST responses (P-value for the trend =0.0004). **(B)** Median OS is significantly improved for patients with RECIST response (P-value for the trend =0.0005).

The median PFS was 70 weeks for patients who had achieved RECIST CR/PR with olaparib therapy, compared to 28 weeks for patients without RECIST CR/PR (log-rank, p=0.0004) (Figure 1A). Median OS was 161 weeks in patients with RECIST CR/PR, compared to 64 weeks in patients who did not achieve RECIST CR/PR (log-rank, p=0.0005) (Figure 1B).

### Platinum sensitivity and antitumor response to olaparib

At the time of enrolment to each trial, 64% of patients were platinum sensitive, while 36% were platinum resistant. Higher likelihood of RECIST CR/PR rates were observed in patients with platinum-sensitive ovarian cancer (35%), in contrast to those with platinum-resistant disease (13%) (p=0.02). Although the RECIST CR/PR rate was significantly lower in platinum-resistant patients compared to those with platinum-sensitive disease (13% versus 35%, p=0.02), 5 of 38 (13%) patients conventionally defined as platinum-resistant still achieved RECIST CR/PR, suggesting that platinum response status is not an absolute predictor of response to olaparib.

### Influence of PTPI on response to olaparib

The median PTPI was 53 weeks (range 4-244 weeks). Median PTPI was significantly longer for patients with platinum-sensitive ovarian cancer (68.7 weeks) compared to patients with platinum-resistant disease (25.9 weeks) (p<0.0001). Independent of their platinum response status, patients with PTPI of greater than 52 weeks had higher CR/PR rates than those with PTPI of less than 52 weeks (42% vs 18%, respectively, p=0.016).

In order to study the association of PTPI with response, we compared the RECIST response rate to olaparib in patients who were classed as platinum-sensitive before commencing olaparib and had PTPI of greater than 24 weeks (‘best-responders’), to patients who were platinum-resistant and had PTPI of less than 24 weeks (‘least-responders’). Fifty-eight patients were defined as ‘best responders’ and 20 patients as ‘least responders’. The RECIST CR/PR rates achieved with olaparib with the ‘best responders’ group were significantly higher than the ‘least responders’ group (34.5% vs 10%, p=0.04). We did not identify any association between response to olaparib and other clinical baseline characteristics, including initial FIGO staging or cytoreductive surgery.

## DISCUSSION

In this international multicenter retrospective study, we investigated the baseline clinical characteristics that may predict for antitumor responses to treatment with the PARP inhibitor olaparib in patients with advanced *BRCA1/2* mutation ovarian cancer.

The hypothesis underlying the concept of synthetic lethality is that patients harboring deleterious germline *BRCA1/2* mutations have homologous recombination deficient tumors, which may be treated effectively with PARP inhibitors [1, 2]. However, despite this, variable response rates to PARP inhibitors have been observed in patients with advanced germline *BRCA1/2* mutation tumors, including those with ovarian cancer [3, 5, 6, 13, 14]. Supplementary Table 1 summarises the different response rates observed in patients with advanced *BRCA1/2* mutation ovarian cancer treated with olaparib in clinical trials. These trials confirmed the impressive activity of olaparib in patients with recurrent *BRCA1/2* mutant ovarian cancers, even in a proportion of patients with platinum-resistant disease [3–7]. A recent pooled analysis of data from six clinical trials with olaparib in BRCA1/2 mutation ovarian cancer patients has confirmed responses in both platinum sensitive and platinum resistant patients, but also describes a category of “platinum status unknown” (response rates in these 3 categories are 48%, 28% and 35% respectively). The authors comment on the challenge of determining platinum status in platinum-resistant patients who receive another non-platinum treatment which may be effective and are then treated with olaparib. [15].

A number of prospective and retrospective studies are currently ongoing to identify molecular biomarkers that may predict antitumor response to olaparib and other PARP inhibitors [8–12, 16]. While prior sensitivity to platinum-based chemotherapy has been reported to predict antitumor responses to olaparib, in our data set, 5 (13%) platinum resistant patients responded to olaparib, suggesting that platinum sensitivity status may not be sufficiently robust as a predictor of response to olaparib. Data from our study suggest that PTPI appears to be an additional clinical indicator of antitumor response to olaparib treatment, which may also be potentially applicable to other PARP inhibitors.

PTPI may be a useful indicator for informing both timing and sequence of non-platinum containing treatment regimens during the treatment journey of patients with advanced *BRCA1/2* mutation ovarian cancer. Our data suggest that patients with a longer platinum-free interval, even after becoming resistant to platinum-based chemotherapies, are more likely to respond to olaparib and potentially other PARP inhibitors. This is consistent with some studies, which have suggested that a longer platinum-free interval may increase antitumor response rates to subsequent treatment, including rechallenging patients with platinum-based therapy [17, 18]. It may therefore be useful to modify the current classification of platinum sensitivity status, which is based on the response to the most recent platinum-based chemotherapy, which could be many months before the patients’ next treatment regimen. While we do not recommend using the PTPI as a sole clinical decision tool, the findings of this study warrants further investigation, ideally prospective validation, to assess the association of PTPI with response to olaparib and other PARP inhibitors in patients with advanced *BRCA1/2* mutation ovarian cancers.

It has previously been speculated that the antitumor response to olaparib may be different in patients who have germline *BRCA1* versus *BRCA2* mutations [19]. In this retrospective study involving 78 patients with germline *BRCA1* mutations and 30 with *BRCA2* mutations, no differences in antitumor response was observed between both groups of patients. Details on the specific *BRCA1/2* gene mutations and their association with response to olaparib were not collected. This requires a larger series of patients that may further explain the lack of antitumor responses to PARP inhibitors in patients with advanced *BRCA1/2* mutation ovarian cancer.

Despite this being one of the largest retrospective series of clinical data from patients with advanced *BRCA1/2* mutation ovarian cancer treated with the PARP inhibitor olaparib, a limitation of this study was the involvement of a relatively small series of patients that prevented further statistical analysis such as regression analysis. Future studies with a larger data set will enable linear analysis to be undertaken to identify a more precise PTPI fit model to predict antitumor response to PARP inhibitors. The patients in our study have mostly undergone several lines of chemotherapy, so it is still possible that there is a population of either *BRCA1* or *BRCA2* patients who are excellent responders to first line chemotherapy and don't need olaparib or alternatively some who are very platinum resistant and don't survive to get olaparib.

The accelerated approval of olaparib by the FDA and EMA in patients with advanced *BRCA1/2* mutation ovarian cancer (without specific platinum sensitivity status, FDA) heralded a new era in precision medicine in patients with advanced ovarian cancer. Several other PARP inhibitors are currently at different stages of clinical development in patients with *BRCA1/2* mutation ovarian cancer. Despite this, improved strategies for selecting patients who may benefit most are urgently required, including clinical factors that can be implemented easily without additional delays, costs or logistical issues. We have shown that PTPI may potentially be an important clinical biomarker that should be considered when treating patients with advanced *BRCA1/2* mutation ovarian cancer with a PARP inhibitor, and warrants further investigation either in conjunction with or independent of conventional platinum response status.

## MATERIALS AND METHODS

Detailed clinical data were collected retrospectively from patient records of 108 patients with advanced recurrent germline *BRCA1/2* mutation ovarian cancer from 8 cancer centers (Royal Marsden Hospital, UK; Edinburgh Centre, UK; University of California San Francisco, USA; Sheba Medical Centre, Israel; Prince of Wales Cancer Centre, Australia; Dana-Farber Cancer Institute, USA; Oncologisch Centrum UZ Brussel, Belgium; Princess Margaret Cancer Centre, Canada), which had participated in clinical trials of olaparib monotherapy between April 2006 and August 2014. This study was approved by the Royal Marsden Clinical Research and Development Committee.

Only patients who had been treated with olaparib at doses of 200 mg tablet or capsule formulation or more twice daily in the relapsed setting were entered to this study. This minimum dose of 200 mg BID was selected on the basis of pharmacodynamic and antitumor activity of olaparib from previous studies, where doses of 200 mg BID resulted in significant target inhibition and antitumor responses [20, 21]. This study did not include any patient who had received olaparib in the maintenance treatment setting.

Baseline patient characteristics were collected and entered into a standardized database; these included details on demographics, surgical debulking, *BRCA1/2* status, prior chemotherapy, platinum-based chemotherapy sensitivity status at the time of enrolment, prior history of breast cancer or treatments received for breast cancer, RECIST and GCIG CA125 responses, survival, response rates and the interval between last platinum-based chemotherapy to the start of olaparib treatment.

Patients were defined as platinum-sensitive or platinum-resistant when the platinum-free interval was more than 12 months or less than 6 months, respectively [22]. Patients with a platinum-free interval of between 6 to 12 months (partially platinum-sensitive) were also classified as platinum-sensitive in this study. Platinum-containing chemotherapy was not necessarily the most recent antitumor treatment that patients had prior to receiving olaparib. We therefore also recorded the time interval between the end of last platinum-containing chemotherapy regimen and the start of olaparib treatment, termed the platinum-to-PARP inhibitor interval (PTPI), as a separate clinical parameter.

Pearson Chi^2^, odds ratios (OR) and Fisher's exact probability tests were used for statistical analyses. Survival curves were generated using GraphPad Prism 6.

## SUPPLEMENTARY MATERIALS FIGURES AND TABLES


