# Supplementary material for: Baseline clinical predictors of antitumor response to the PARP inhibitor olaparib in germline BRCA1/2 mutated patients with advanced ovarian cancer
Source: Oncotarget. 2017 Apr 10;8(29):47154–60. doi: 10.18632/oncotarget.17005 (PMC5564551; doi:10.18632/oncotarget.17005)
Supplement: Supplementary file 1 [file oncotarget-08-47154-s001.pdf]

Baseline clinical predictors of antitumor response to the PARP inhibitor olaparib in germline BRCA1/2 mutated patients with advanced ovarian cancer

SUPPLEMENTARY TABLE

Supplementary Table 1: Response rates to olaparib in patients with advanced *BRCA1/2* mutation ovarian cancer

Trial	No. of patients	Olaparib dose	RECIST and/or CA125 response		
			Overall	Platinum sensitive	Platinum resistant
**Phase I, Fong et al, 2010**	50	39 pts, 200mg BID<br>11 pts, dose escalation	40%	61.5%	41.7%
**Phase II, Audeh et al, 2010**	57	33 pts, 400mg BID	33%	38%	30%
		24 pts, 100mg BID	12.5%	50%	0
**Phase II, Kaye et al, 2011**	64	32 pts, 400mg BID	59%	-	-
		32 pts, 200mg BID	38%		
**Phase II, Gelmon et al, 2011**	17	400mg BID	41%	60%	33%
**Phase II, Kaufman et al, 2015**	193	400mg BID	-	-	31%
